# Supplementary material for: Cross border population movements across three East African states: Implications for disease surveillance and response
Source: PLOS Glob Public Health. 2024 Oct 16;4(10):e0002983. doi: 10.1371/journal.pgph.0002983 (PMC11482666; doi:10.1371/journal.pgph.0002983)
Supplement: Attachment — Submitted filename: Response to reviewers_ PopCAB_9_6_24.docx [file pgph.0002983.s002.docx]

Greetings,

We would like thank the reviewers for their comments and suggestions, this is a point-by-point response to the reviewer comments;

**Reviewer #1**:

1. The is no association of public health factors with population movements

Response: **We acknowledge the importance of linking public health to movement in this paper we have highlighted public health factors linked with population movements including: Travel related health seeking behavior for both conventional and traditional medical services and perceptions towards disease cross border disease surveillance. We further discuss how economic and social related travel may influence disease transmission.** Refer to line 229-234

2. The times and periods when the movements are at peak or lowest are not clearly specified even though factors associated with the movements are indicated

**Response:** This has been addressed, we have included movement peak peaks, refer to line 294-304

The peak times of travel were related to agricultural seasons, religious and cultural festivals.

“… From Uganda to Kenya movement is mainly January - April and June – August during cultivating and planting seasons… From Kenya to Uganda in December during cultural festivals…they generally move throughout the year but the months mentioned above have the most movements…”FDG P3 Lwakhakha

“… travelers from Rwanda move to Uganda in June to look for jobs in Mubende district during the harvest season…In December they travel back to celebrate Christmas with their families….” KII Cyanika

“…..Ugandans travel to Rwanda to Celebrate the new year’s day… In Rwanda they celebrate better than in Uganda……” KII Cyanika

3. It reads like the authors picked a section from a larger data set because this paper would be stronger if it had some quantitative data on surveillance that would include disease outbreaks and epidemics and how they impact population movement

Response: **We have included a section on the influence of EVD outbreak on movement patterns. Refer to line 316-333**

**Influence of ongoing EVD outbreak in Uganda on population movement**

Respondents reported that influence of the ongoing EVD outbreak in Uganda was different for each country. There was reduced movement to Uganda from Rwanda while movements cross the Uganda-Kenya border were not affected.

“…Ebola outbreak has led the reduction of movements to Uganda because Rwanda has put more restrictions for travellers returning from Uganda... a traveller has to be tested every time he/she crosses border…security guards have been deployed along porous borders; and this movements reduction has caused total loss in business…”

“…. people have continued to go about their business but with caution because we are at the border and anything is bound to happen

Some cross border communities did not perceive EVD as a threat and therefore carried on with travel was to be very far from them; in Kampala and Mubende with no reason for worry. They reported that EVD had not scared them as much as coronavirus did.

“…The fact that Ebola has not yet reached Nyabitare village, it has not compromised people’s movements across borders… some youths still travel to Mubende, an active outbreak area in search fro jobs in Agriculture…”

“…Some business men and truck drivers continue to travel to markets in Jinja and stay there for a couple of days during the ongoing EVD outbreak….”

4. I believe there should be a section on policy and governance and how it impacts population movements

Response: **We acknowledge the importance of policy and governance and its impact on population movement; however, this was not included in the scope of our study and we did not collect data on this.**

5. The authors should provide a brief descriptive description of the study participants and how they were distributed, for example, how many were from Kenya, Uganda or Rwanda

**Response: We have included a brief distribution on the participants in the FDGs and KIIs. Refer to line 114-126**

6. Other than the mapping, the authors should specify the geographical factors that attract migrants/ mobile populations to specific sites.

Response**: We have added information on geographical factors that influence movement ref to line 254-261**

**Geographical factors**

Participants reported several geographic factors influencing movement, these include mineral resources, topographical features, plants and animals in national parks and reserves.

“…tourists move to see the mountain caves at Mgahinga National Park, the highest peak of the three mountains which is Muhabura Mountain and Bwindi impenetrable national park…” KII Cyanika

“…travelers move to Mubende from Rwanda for gold mining….” P7, FGD Cyanika

7. I believe recommendations are not strong because critical data/information on public health is lacking

Response: **We have revised recommendations refer to line 418-423**

We recommend enhanced community-based surveillance in identified key high-risk areas such as markets, key destinations and porous borders. Behavioral change communication interventions targeted at risky behavior tailored to the needs of cross border communities and tran national coordination and service delivery of HIV treatment services could improve continuity of care in cross border communities.

**Reviewer #2:**

**Weakness of the Manuscript**

The weakness of the manuscripts relies mainly on the connection of the population movement and the spread of the diseases in question. This should be explained further. It is not clear which questions were asked and what responses were provided in relation to for instance EVD.

**Response:** Questions asked in relation to EVD have been added to the manuscript on line 128- 129

“Influence of public health threats like EVD on movement, recent movement to EVD affected areas”

Secondly, the use of grounded theory is important for this study but the authors seem not to explain in depth how this was utilized, issues of open coding, themes/categories, axial coding for making sense of data, and selective coding to help in the development of draft theory should be well elucidated.

**Response**: **This was an oversight in the abstract, we used “thematic analysis” this has been corrected**

**Major Issues**

a) The title needs refocusing: Cross Border Population Movement Patterns is quite broad. For clarity: It is not clear in the title what this cross-border population movement relates to. The authors need to revisit the title to reflect what is written in the main text of the manuscript. Is November 2022 important to be included in the title?

**Response:** We have revised the title to capture the content of the manuscript– “Cross Border Population Movements across 3 East African States: Implications for Disease Surveillance and Response, 2022”

b) FGDs for business persons- it is not clear whether these were separated by gender (Male, female). Please explain what was done since the authors indicated that for the other categories of FGDs the participants were male but for the business person they included females as well.. If the GFDs were disaggregated by gender it would be important to know what were the key issues emanating from the female FGDs compared to the male FGDs,

Response: We have revised to clarify this in the manuscript - FDGs for business persons and travelers were disaggregated by gender

c) Ethics: It is indicated that the study was reviewed by the United States Centers for Disease Control and Prevention (U.S CDC) human subjects review board and consistent with applicable federal law and CDC policy. What about REC from Uganda/Kenya/Rwanda? And for Uganda was the study submitted to UNCST for approval from the UNCST? Or this was unnecessary.

Response: **The study was done in reference to a memorandum of understanding and umbrella protocol (Grant number: NU14GH001238) between the field Epidemiology training programs of the three countries (Uganda, Kenya, Rwanda), this study was waived the process of full institutional review board.**

d) In the discussion section there is a lot of writeup on EVD outbreaks presenting a potential risk of transmission of EVD to these countries. However, it is not well presented in the findings. Please revisit the transcripts (FGDS and KII) to get some quotes to give us the voices of the participants related to their concerns about such diseases. But their lack of concern is also a finding.

Response: This has ben addressed, more information on EVD has been added refer to line 308-325

**Minor issues**

Several typos are evident and these need to be addressed. Examples include the abbreviation of focus group discussions is FGD. But in some cases, the authors abbreviate it as FDG.

Response: **These have been revised this.**
